# Supplementary material for: Fluid extraction from the left-right organizer uncovers mechanical properties needed for symmetry breaking
Source: eLife. 2023 Jul 21;12:e83861. doi: 10.7554/eLife.83861 (PMC10361723; doi:10.7554/eLife.83861)
Supplement: Supplementary file 1. — Analyses of cilia motility per LRO anterior and posterior halves. [file elife-83861-supp1.docx]

| Sham | Embryos | Anterior | | Posterior | | Total motile cilia | Total cilia |
| --- | --- | --- | --- | --- | --- | --- | --- |
|  |  | Motile | Immotile | Motile | Immotile |  |  |
|  | E1 | 32 | 2 | 18 | 2 | 50 | 54 |
|  | E2 | 20 | 7 | 15 | 8 | 35 | 50 |
|  | E3 | 11 | 4 | 10 | 5 | 21 | 30 |
|  | E4 | 18 | 1 | 11 | 0 | 29 | 30 |
|  | E5 | 17 | 3 | 12 | 2 | 29 | 34 |
|  | E6 | 30 | 1 | 16 | 7 | 46 | 54 |
|  | E7 | 14 | 7 | 14 | 3 | 28 | 38 |
| No LR Defects | E1 | 22 | 3 | 16 | 8 | 38 | 49 |
|  | E2 | 11 | 7 | 10 | 6 | 21 | 34 |
|  | E3 | 19 | 2 | 12 | 4 | 31 | 37 |
|  | E4 | 21 | 2 | 24 | 5 | 45 | 52 |
|  | E5 | 32 | 3 | 24 | 2 | 56 | 61 |
|  | E6 | 11 | 13 | 8 | 7 | 19 | 39 |
| LR Defects | E1 | 3 | 19 | 3 | 22 | 6 | 47 |
|  | E2 | 15 | 10 | 12 | 3 | 27 | 40 |
